# Omnidirectional color filters capitalizing on a nano-resonator of Ag-TiO_2_-Ag integrated with a phase compensating dielectric overlay

**DOI:** 10.1038/srep08467

**Published:** 2015-02-16

**Authors:** Chul-Soon Park, Vivek Raj Shrestha, Sang-Shin Lee, Eun-Soo Kim, Duk-Yong Choi

**Affiliations:** 1 Department of Electronic Engineering, Kwangwoon University, 20 Kwangwoon-ro, Nowon-gu, Seoul 139-701, South Korea; 2 Laser Physics Centre, Research School of Physics and Engineering, Australian National University, Canberra, ACT 2601, Australia

## Abstract

We present a highly efficient omnidirectional color filter that takes advantage of an Ag-TiO_2_-Ag nano-resonator integrated with a phase-compensating TiO_2_ overlay. The dielectric overlay substantially improves the angular sensitivity by appropriately compensating for the phase pertaining to the structure and suppresses unwanted optical reflection so as to elevate the transmission efficiency. The filter is thoroughly designed, and it is analyzed in terms of its reflection, optical admittance, and phase shift, thereby highlighting the origin of the omnidirectional resonance leading to angle-invariant characteristics. The polarization dependence of the filter is explored, specifically with respect to the incident angle, by performing experiments as well as by providing the relevant theoretical explanation. We could succeed in demonstrating the omnidirectional resonance for the incident angles ranging to up to 70°, over which the center wavelength is shifted by below 3.5% and the peak transmission efficiency is slightly degraded from 69%. The proposed filters incorporate a simple multi-layered structure and are expected to be utilized as tri-color pixels for applications that include image sensors and display devices. These devices are expected to allow good scalability, not requiring complex lithographic processes.

Nano-structural color filters have been extensively researched as crucial elements for a variety of applications, such as for display/imaging devices, organic light emitting diodes, organic solar cells, etc^1^,^2^,^3^,^4^,^5^. These are regarded to be a promising alternative to conventional colorant pigment based filters that are vulnerable to optical damage and are somewhat hazardous to the environment. So far, various types of color filters have been developed, such as those based on subwavelength gratings, nano-resonators, plasmonic nanostructures, photonic crystals, and multilayer thin-films^6^,^7^,^8^,^9^,^10^,^11^. However, these have been inevitably limited in terms of their practical implementations since they present angle-sensitive spectral characteristics that manifest as a spectral shift of the center wavelength and a degradation of the transmission/reflection efficiency of the oblique incidence. For some of nanophotonic devices, including metallic nano-antenna array and plasmonic nano-structures^12^,^13^,^14^,^15^,^16^, many attempts have been made to mitigate their angular sensitivity. However, the issue of scalability still remains to be tackled due to the inevitable use of sophisticated fabrication techniques such as e-beam or nano-imprint lithography^13^,^14^,^15^,^16^. A highly scalable filter device should be preferably constructed without resting on multiple steps of lithography^6^,^7^,^8^,^9^,^10^,^11^. In order to develop angle-invariant color filters at a large scale, configurations that resort to the use of a high-index cavity, made of amorphous silicon (a-Si), have been particularly suggested^17^,^18^. Despite the accomplishments of having a notable angle-insensitive performance, to a certain extent, the embodiment of a tri-color set uniformly offering a high efficiency has been severely hindered by the inherently high optical absorption of an a-Si cavity in the visible band. Meanwhile, metal-dielectric (MD) layered structures, which have been actively adopted in various photonic applications including optical filters, are known to potentially offer a remarkable resonant transmission^19^,^20^,^21^,^22^,^23^,^24^,^25^,^26^. In particular, metal-dielectric-metal (MDM) structures based on a Fabry-Perot etalon, exhibiting an efficient spectral window, have been previously proposed to realize various functional devices like color filters^18^,^24^,^25^,^26^,^27^. The property of omnidirectional resonance (ODR) is integral to such color filters, so as to enable angle-invariant performance, in addition to properties such as high efficiency and flexible tuning of the center wavelength. The ODR could be achieved by properly adjusting geometrical parameters for a given material combination^24^,^25^,^26^ and was only realized at a unique and fixed wavelength^24^, thereby making the approaches not suitable for the color filters, where the resonant wavelengths should be tailored across the three primary color bands.

In this work, we realize the ODR over a broad spectral band encompassing the three primary colors, by tapping into a dielectric overlay on an MDM structure, which plays a dual-role of providing appropriate phase compensation and suppressing unwanted reflection. We specifically proposed and demonstrated omnidirectional transmission-type color filters tapping into an Ag-TiO_2_-Ag nano-resonator with an integrated dielectric TiO_2_ overlay, giving rise to highly efficient, angle-invariant spectral responses. The dielectric overlay, acting as a phase compensating layer, is deemed to play a dual-role by substantially de-escalating the angle-sensitive characteristics and suppressing the undesired reflection. We have keenly explored the influence of the overlay upon the ODR, leading to angle-invariant characteristics. The sensitivity of the filter to the polarization is also investigated with respect to the incident angle. Silver (Ag) is specifically chosen as the metallic layer due to its low extinction coefficient and its absence of inter-band transitions that cause an optical loss in the visible band^28^, and TiO_2_ is reserved for use in both the cavity and the phase-compensating overlay as a result of its low loss and relatively high refractive index. Ag and TiO_2_ can be successively deposited in a sputtering chamber, enabling a cost-effective and simple fabrication process.

## Results

### Omnidirectional color filters based on a nano-resonator incorporating a dielectric overlay

**Figure 1:**
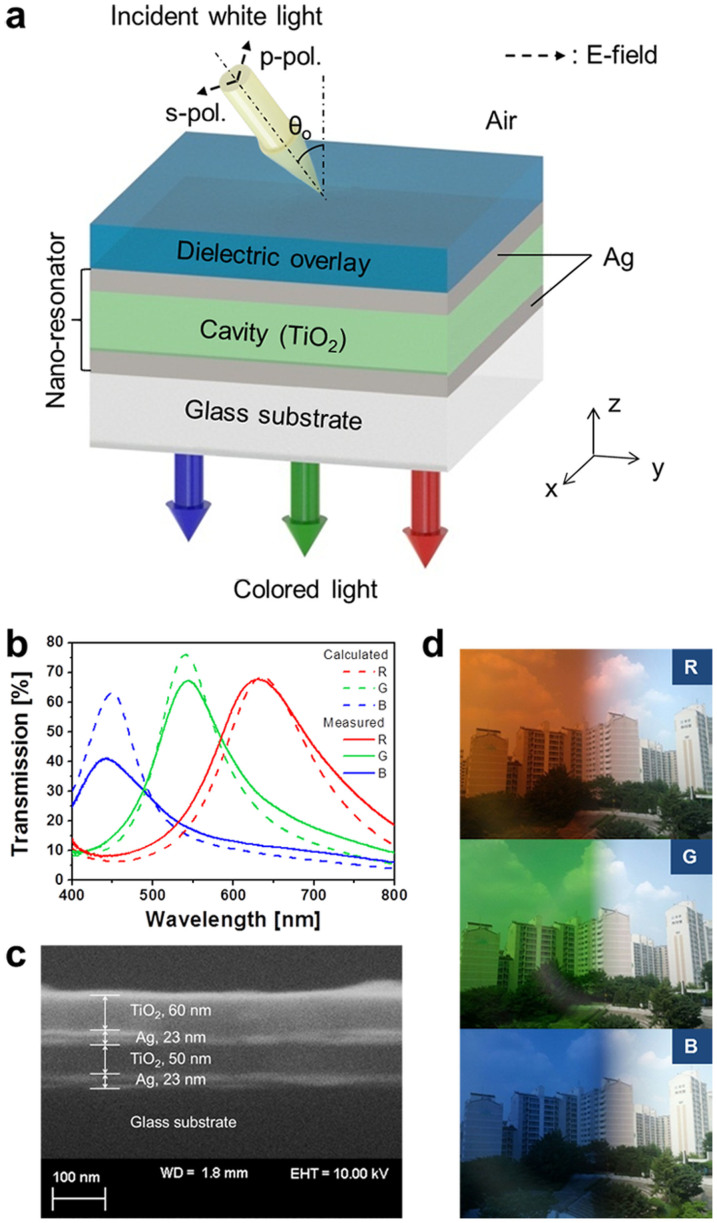
Proposed omnidirectional color filters based on a nano-resonator integrated with a dielectric overlay. (a) Schematic configuration. (b) Simulated and measured optical responses in the range of 400 to 800 nm. (c) SEM images of the prepared device showing the constituent Ag and TiO_2_ layers. (d) Photographic images captured through the RGB filters, presenting bright and vivid colors, taken in Kwangwoon University by C.S. Park.

As depicted in Figure 1(a), the proposed transmission-type color filter consists of a metallic nano-resonator that is combined with a dielectric TiO_2_ overlay, which incorporates a TiO_2_ cavity sandwiched between two ultra-thin Ag films. The nano-resonator mimicks an MDM Fabry-Perot etalon and is basically presumed to exhibit bandpass filtering properties^29^. The dielectric overlay atop the resonator plays the dual-role of enhancing the angular tolerance as well as the transmission efficiency, as will be discussed later. With respect to the light impinging upon the filter, the incident angle is denoted as θ_o_, and the polarization is denoted as s (TE) and p (TM), in accordance with the electric field that is aligned perpendicular and parallel to the plane of incidence, respectively, as indicated in Figure 1(a). The color filters have been adequately designed and manufactured with a footprint of 7.63 × 2.54 cm^2^ by alternately depositing Ag and TiO_2_ layers on a glass substrate via RF sputtering. Each of the Ag layers is 23 nm thick, and the TiO_2_ cavity is determined to be 100, 75, and 50 nm thick, so as to obtain peak transmission at resonant wavelengths of λ_o_ = 636, 541, and 450 nm, respectively corresponding to the red (R), green (G), and blue (B) spectral bands. The refractive indices of the materials used in the simulations are given in Supplementary Figure S1. Figure 1(b) shows the transmission spectra that were calculated and measured for the normal incidence, providing transmission efficiencies that reach up to 69%. The image obtained through a scanning electron microscope (SEM) for the blue filter that was prepared is shown in Figure 1(c), proving a high fidelity to the design. Finally, vivid and bright images taken using the fabricated RGB filters are displayed in Figure 1(d).

**Figure 2:**
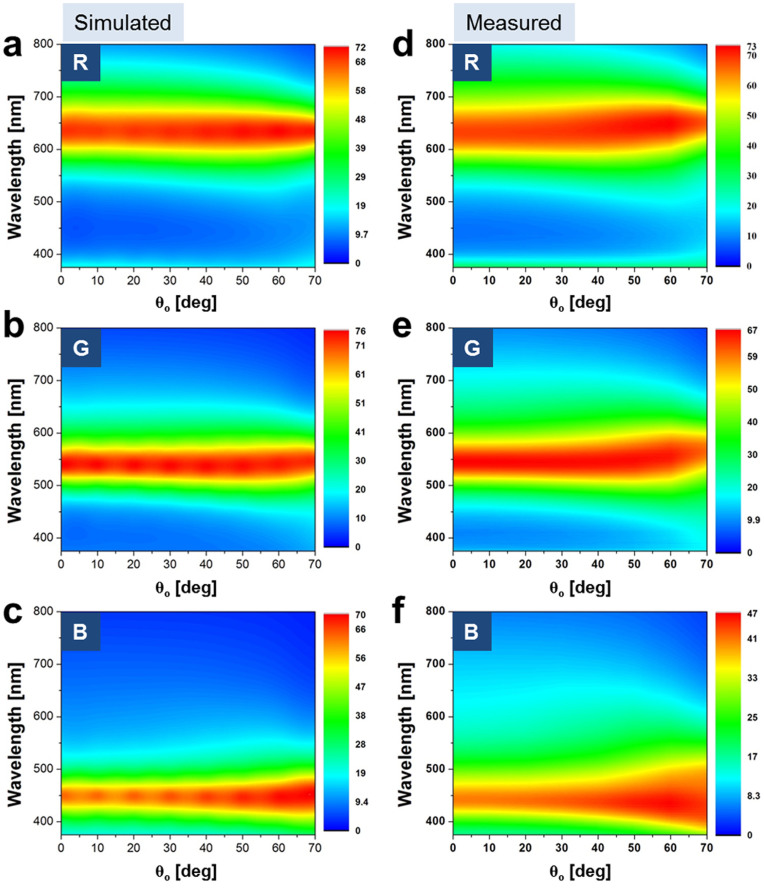
Transmission spectra for incident p-polarized light. (a–c) Contour maps of calculated transmission spectra for incident p-polarized light for the RGB filters. (d–f) Contour maps of the measured transmission spectra for p-polarized incident light for the RGB filters.

**Figure 3:**
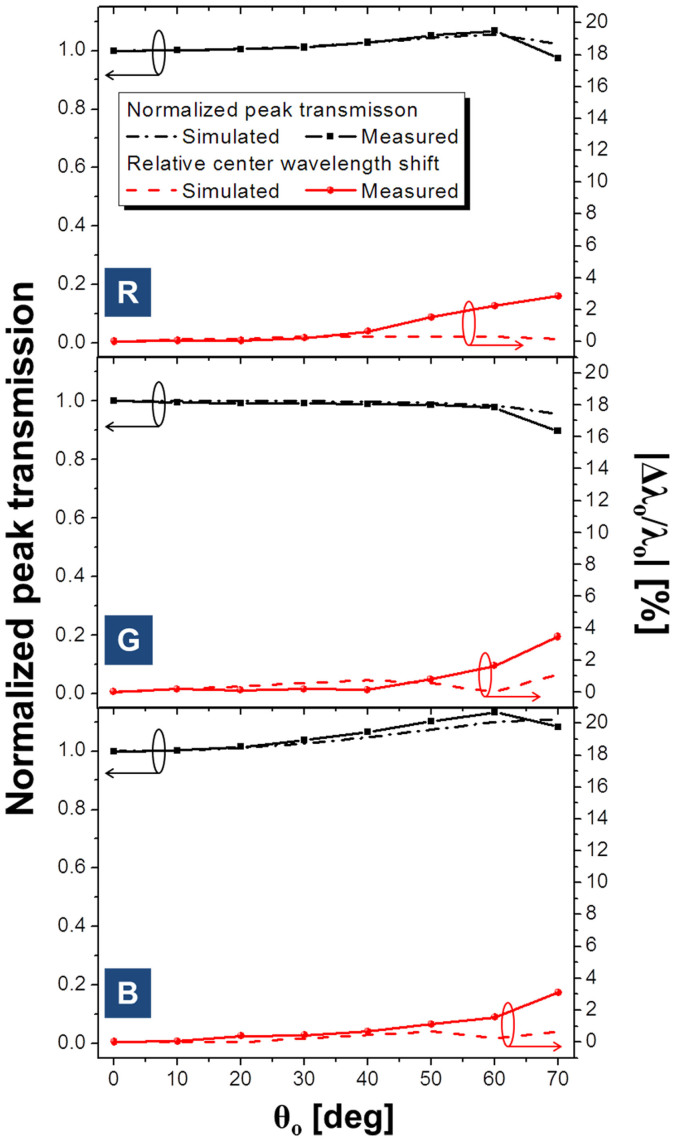
Angular performance of the filters for p-polarized light. Simulated and measured relative peak transmissions, as indicated with dashed and solid black lines, respectively, and the corresponding shifts in the relative center wavelength marked in dashed and solid red lines, respectively, for the red, green, and blue colors, from top to bottom.

We first investigate the angular dependency of the transfer characteristics of the filters for p-polarized incident light. Figure 2 shows contour maps pertaining to the calculated and the measured transmission spectra, with an incident angle ranging from 0° to 70°. Good correlations are observed between results from the simulation and the measurement. The RGB filters have transmission peaks that are apparently maintained at λ_o_ = ~636, 541, and 450 nm, regardless of variations in the incident angle. In a bid to better understand the angular sensitivity of the filters, the transfer characteristics are assessed in terms of the changes in the normalized peak transmission as well as shift in the relative center wavelength, defined as |Δλ_o_/λ_o_|, where λ_o_ is the resonant wavelength and Δλ_o_ is the spectral deviation from the center wavelength corresponding to the normal incidence. Figure 3 plots the normalized peak transmission and the shift in the relative center wavelength for p-polarized light through the tri-color filters, as the incident angle changes from θ_o_ = 0 to 70°. The actual shifts in the wavelength Δλ_o_ for the RGB filters are about 18.0, 18.9, and 13.7 nm for θ_o_ = 70°, translating into |Δλ_o_/λ_o_| of ~2.8, 3.5, and 3.1%, respectively. The degradation in the normalized peak transmission is less than 0.5 dB throughout the entire range of θ_o_. The transmission for certain cases with oblique incidence is notably found to surpass the transmission for the case where θ_o_ = 0°. This may be attributed to the fact that the reflectivity for p-polarized light tends to decrease as θ_o_ increases up to the Brewster angle, incurring no reflection, and then it increases for angles beyond that angle^30^. A discrepancy between the calculated and the measured results is imputed to the partial reflection from the glass-air interface at the bottom of the substrate, which sharply increases as θ_o_ increases, which is not taken into account during the calculations.

In order to validate the effect of the dielectric overlay, both theoretical and experimental comparison between the cases with and without the overlay have been performed in terms of the normalized peak transmission and relative center wavelength shift (|Δλ_o_/λ_o_|) for green filters. The green filters based on (Ag-TiO_2_-Ag)|TiO_2_ and Ag-TiO_2_-Ag provide transmission efficiencies of 67 and 45% for the normal incident light, respectively. As shown in Supplementary Figure S2, for the filter without the TiO_2_ overlay, the center wavelength is simulated to shift by as much as 40 nm, translating into |Δλ_o_/λ_o_| of ~5.9%, when θ_o_ varies from 0 to 70°. The absolute and relative wavelength shifts are measured to be 54 nm and 9.2%, respectively. For the same range of θ_o_, the calculated and measured peak transmissions remain almost constant for the device of (Ag-TiO_2_-Ag)|TiO_2_ configuration, while the transmission increases slightly with increasing θ_o_ for the case without the TiO_2_ overlay. The transmission efficiency for the device without the overlay is inclined to rise slightly with θ_o_, yet below the level corresponding to the structure loaded with the overlay. It should be remarked that the transmission is substantially less sensitive to the angle for the case with the overlay as compared to the case without the overlay, as will be discussed later. As a consequence, it has been confirmed both theoretically and experimentally that the dielectric overlay helps improve the angle invariant performance with respect to the peak transmission and center wavelength shift.

### Role of the phase-compensating dielectric overlay and the mechanism behind the ODR

**Figure 4:**
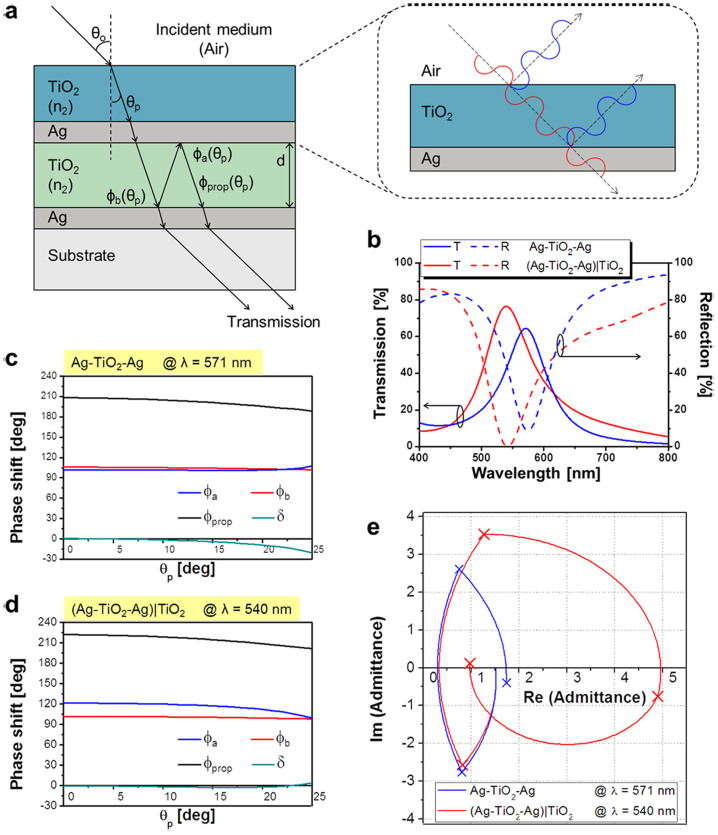
Effect of the phase compensating TiO_2_ overlay leading to both AR and ODR. (a) Ag-TiO_2_-Ag nano-resonator incorporating a phase compensating overlay consisting of a 60-nm thick TiO_2_ film. (b) Transfer curves for the transmission/reflection spectra of the structure without and with the overlay. Total phase difference for the structure (c) without and (d) with the overlay. (e) Admittance diagram for the nano-resonator structure without and with the overlay.

The proposed filter is systematically characterized in terms of the reflection, phase shift, and optical admittance as depicted in Figure 4, in order to examine the role of the dielectric overlay in tandem with the nano-resonator in attaining the ODR, which is closely associated with the angular invariance and concurrent suppression of the reflection over the visible spectral range. As shown in Figure 4(a), the light originating from the air at an angle of θ_o_ is initially refracted into the dielectric overlay at an angle of θ_p_, in compliance with the Snell's law^31^. Inside of the TiO_2_ cavity, light preserves the same angle of propagation as θ_p_. The transmission/reflection spectra depending on the adoption of dielectric overlay are plotted in Figure 4(b). The presence of the TiO_2_ overlay increases the peak transmission from 64.3 to 76.5% (from the solid blue curve to the solid red curve) while diminishing the reflection from 9.2 down to 0.4% (from the dashed blue curve to the dashed red curve), thereby validating that the overlay is an effective anti-reflection (AR) coating. Both the transmission and reflection spectra undergo a blue-shift in the center wavelength from 571 to 540 nm, and the role of the overlay as an AR coating will be discussed later.

Next, we investigate the requirements to achieve ODR in an MDM nano-resonator with an optical power transmission that is essentially given as 
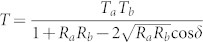
, where R_a_ and R_b_ are, respectively, the reflectivities of the top and bottom Ag-TiO_2_ cavity interfaces, and T_a_ and T_b_ are the transmissivities through the interfaces^31^. The total phase accumulated during a single round-trip within the cavity is given as *δ* = *ϕ_prop_* − (*ϕ_a_* + *ϕ_b_*), where *ϕ_a_* and *ϕ_b_* are the phase shifts imparted by the internal reflection at the top and bottom Ag-TiO_2_ cavity interfaces. The round-trip propagation phase shift is given as *ϕ_prop_* = (4*π/λ*)*n*_2_*d*cos*θ_p_*. Here, n_2_ and d respectively represent the refractive index and the thickness of the cavity, with a transmission maximum occurring at a resonant wavelength λ_o_ for a phase difference of *δ* = 2*mπ*, with m being an integer^31^. The above discussions reveal that the resonant wavelength can be kept constant irrespective of θ_o_. Therefore, a high level of ODR can be achieved as long as *δ* is preserved as integer multiples of 2π (or 360°). Thus, it should be clear at this point that in order to achieve ODR, the round-trip propagation phase shift and the reflection phase shifts at the top and bottom Ag-cavity boundaries are altogether required to sum up in such a way that eventually the total phase shift becomes integer multiples of 2π.

Thus, we have thoroughly investigated the core mechanism underlying the ODR of the proposed angle-invariant color filter exploiting a nano-resonator, by performing detailed observations of the contributions of different types of phase shifts associated with the cavity. Figures 4(c) and 4(d) respectively present the phase shifts for the cases for a typical green filter at different propagation angles θ_p_ in the cavity without and with the overlay. Note that θ_p_ is equivalent to ~25° at λ = 540 nm for θ_o_ = 70°, as predicted by the Snell's law. Accordingly the propagation angle within the cavity is merely supposed to change from 0° to 25°, as shown in Figures 4(c) and (d). The detailed results for the two different types of tri-color filters are presented in Supplementary Figures S3 and S4. As seen in Figure 4(c), the accumulated phase shift *δ* that results from the round-trip propagation as well as the reflection at the top and bottom interfaces of the cavity changes with θ_o_, causing a shift in the resonant wavelength. However, when a 60-nm thick TiO_2_ overlay is introduced in the Ag-TiO_2_-Ag nano-resonator, *δ* is kept almost constant at 0 since the phase shift for the reflection at the top interface is perfectly compensated for by the angle-dependent propagation phase, as implied in Figure 4(d). Consequently, we could achieve the desired ODR, incurring minimal variations in the resonant wavelength associated with the incident angle. Despite variations in θ_o_, the dielectric overlay is verified to successfully serve as a phase compensating layer, which helps maintain the phase shift transpiring through the nano-resonator and substantially alleviate the angle-dependent characteristics of the filter.

Moreover, in order to gain further insight into the use of the dielectric overlay as an AR coating, we characterize the proposed structure by means of an optical admittance diagram. As illustrated in Figure 4(e), the admittance that corresponds to the center wavelength for the transmission/reflection spectra is traced for the nano-resonator without and with the overlay. The effective admittance Y that corresponds to the entire structure, as observed with reference to the incident medium, is expressed as Y = x + jy, where x and y, respectively, are real and imaginary parts of the admittance. The reflection is known to be determined by 
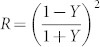
, assuming that air is the incident medium. In order to minimize the reflectance and maximize the transmission efficiency, we preferably need to diminish the difference between the effective admittance of the entire multi-layer structure and the admittance of the incident medium (the air), equivalent to (1, 0)^18^. Figure 4(e) indicates that, as a result of the dielectric overlay, the effective optical admittance, as observed from the top of the multi-layered structure, shifts from (1.74, −0.40) for the Ag-TiO_2_-Ag case to (0.98, 0.12) for the (Ag-TiO_2_-Ag)|TiO_2_ case including the overlay. Thus, it approaches that of air. As a result, the reflection is reduced and the transmission increases, as shown in Figure 4(b). In particular, the overlay made of a 60-nm thick TiO_2_ (n = ~2.22 at λ = 540 nm) film is predicted to almost account for a quarter wavelength. As illustrated in the magnified portion of the structure seen in Figure 4(a), the round-trip phase shift for the film amounts to about 180°, so destructive interference between the reflected waves takes place and improves the transmission efficiency. It should be noted that for the overlay serving as an AR coating, the optimum thickness for the green filter, with a center wavelength around 540 nm, is about 60 nm, while the corresponding thicknesses for the red and blue filters are about 48 and 72 nm, respectively. Noting that the overlay is also responsible for the phase compensation relevant to the center wavelength shift that is induced by θ_o_, we have examined the effect of the thickness of the overlay on the performance of the color filters in terms of the transmission and |Δλ_o_/λ_o_| with θ_o_, as shown in Supplementary Figure S5. It is observed that an enhanced angular insensitivity incurring minimal center wavelength shifts, leading to satisfactory ODR, is concurrently achieved for all of the three color filters, sharing in the same thickness of 60 nm. As a result of using a uniform 60-nm thickness for the dielectric overlay, the transmission is expected to just decline by less than 7% and the center wavelength appears to be kept nearly constant, when θ_o_ varies up to 70° for both red and blue filters, compared to the case of using the corresponding optimal AR thickness. Therefore, the overlay thickness for the tri-color filters is chosen to be the same at 60 nm, taking into account the fact that from the viewpoint of convenience of device fabrication the tri-color pixels could be made without requiring additional lithography processes.

### Influence of light polarization in terms of the incident angle upon the performance of color filters

**Figure 5:**
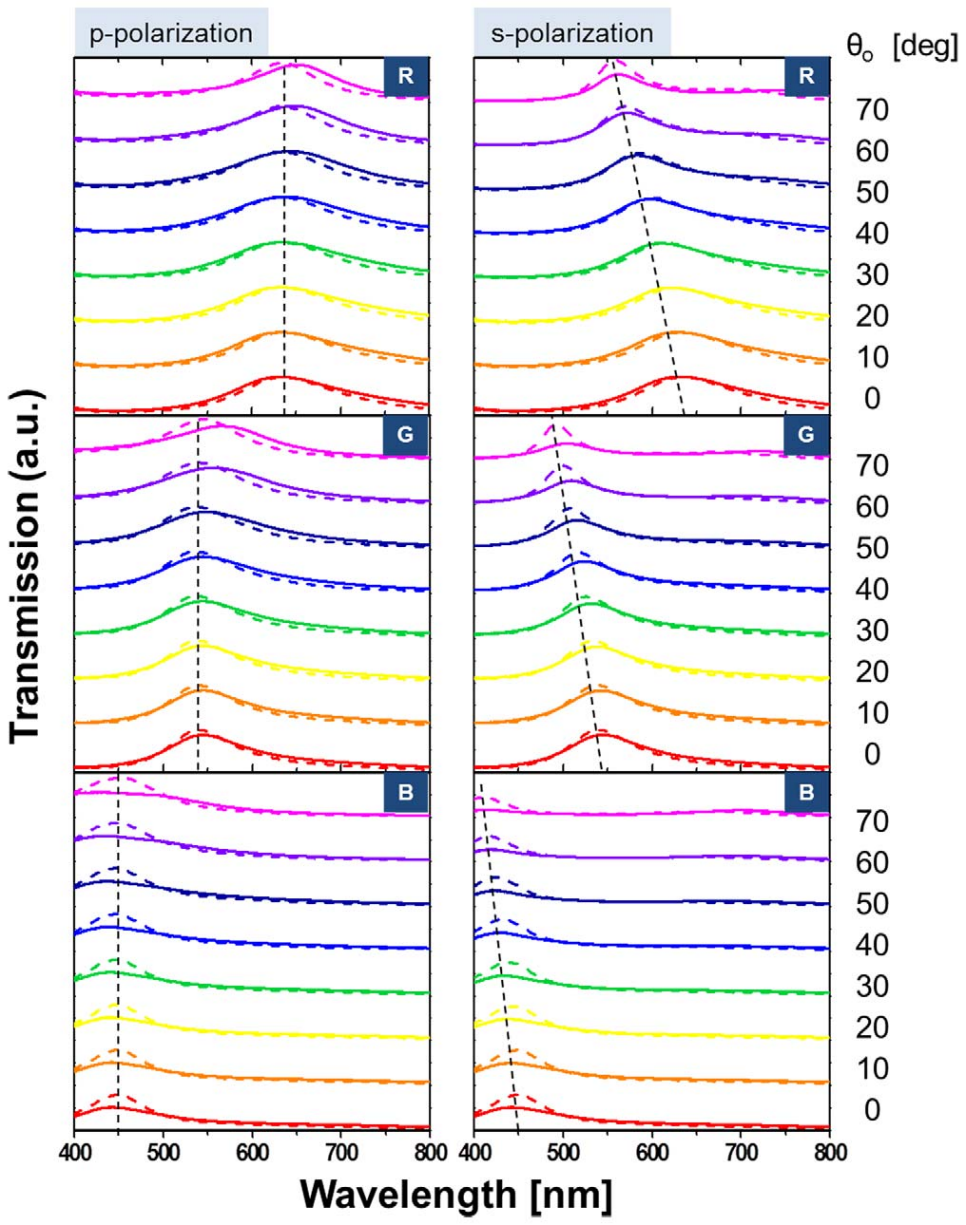
Transmission spectra at different incident angles. Calculated (dashed) and measured (solid) transfer characteristics for the RGB filters for p-polarized (left) and s-polarized incident light (right).

**Figure 6:**
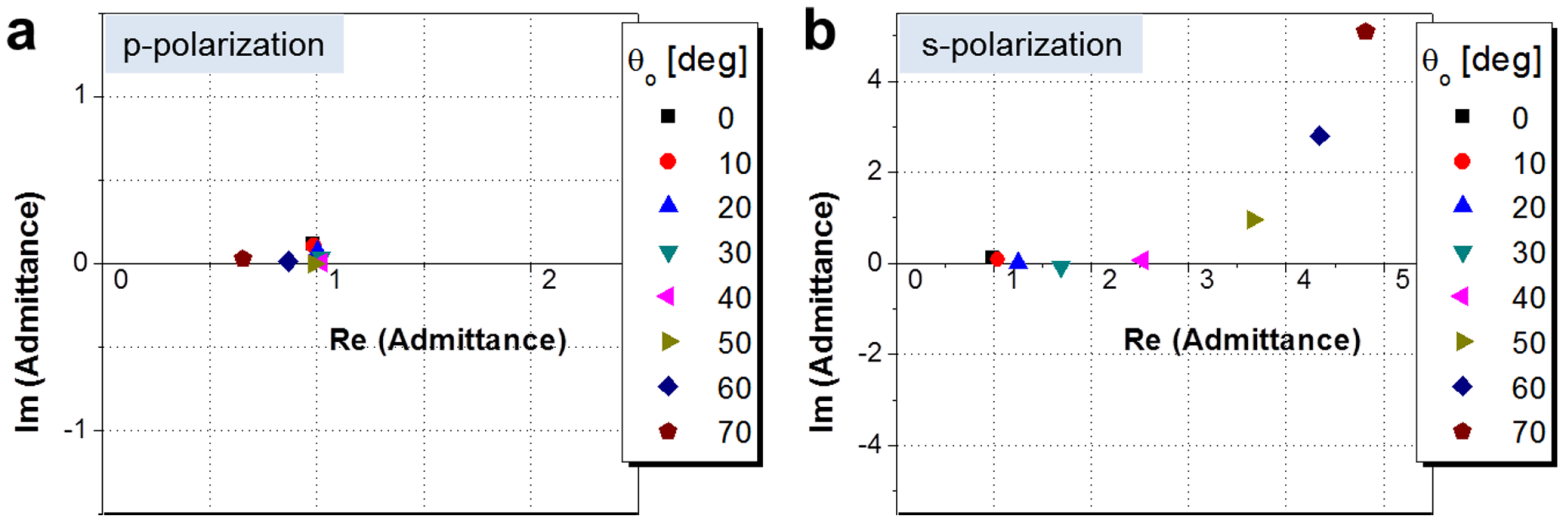
Calculated admittance corresponding to the filter structure in terms of the incident angle for (a) p-polarization and (b) s-polarization.

In an effort to elucidate the impact of the polarization of light upon the angular dependence, as shown in Figure 5, we checked the calculated and measured transmission spectra for the p- and s-polarizations at different incident angles. It seems that the shift in the center wavelength with respect to θ_o_ is more pronounced for the s-polarization than for the p-polarization. For an oblique incidence, with angles taking up to 70°, the shifts in the center wavelength are below ~18.9 and 71.4 nm for the p- and s-polarization, respectively. Taking into account the fact that the transmission can be evaluated via a system of cascaded thin films by virtue of the transfer matrix method, which engages the admittance of each film, we have scrutinized the admittance of the filters for cases with oblique incidence, and we have found the angular dependence in terms of the polarization^18^,^30^. The tilted optical admittance of a thin film in cases with oblique incidence for p- and s-polarization is given by η_p_ = Ycosθ_o_/cosθ and η_s_ = Ycosθ/cosθ_o_, respectively, where Y is the optical admittance, θ_o_ is the angle in the incident medium, and θ is the propagation angle inside of the film of concern^30^. The tilted admittance is discovered to more sharply increase as θ_o_ increases for the s-polarization than for the p-polarization, which stems from its respective dependence on cosθ_o_ and 1/cosθ_o_. As a result, the difference in the admittance for the two polarizations increases with larger incident angles. In order to validate this observation, we calculated the admittance Y(x, y) for the entire structure for various incident angles, as depicted in Figure 6. The admittance is seen to remain fairly constant for the p-polarization, but it varies severely for the s-polarization. Hence, unequal angular transfer characteristics that hinge on the polarization are accordingly ascribed to different evolutions in the admittance. Furthermore, the total phase shift turns out to exhibit different angular dependencies for the two polarizations (Supplementary Figure S4), leading to a polarization-sensitive shift in the resonant wavelength for the transmission/reflection spectra for different incident angles. With respect to the center wavelength shift, substantial angle-invariant characteristics were witnessed for p-polarization as compared to those for s-polarization.

## Discussion

We present a set of omnidirectional color filters that utilize an Ag-TiO_2_-Ag nano-resonator integrated with a dielectric overlay, giving rise to a high transmission efficiency that reaches up to 69% with acceptance angles ranging up to 70°. The TiO_2_ cavity was designed to have a thickness of 100, 75 and 50 nm, in order to produce vivid red, green and blue colors, respectively. The dielectric overlay was proven to serve the dual-role of lessening the angular dependence of the transfer characteristics and of boosting the transmission as an AR coating. For the overlay, we have selected the same material as the cavity, TiO_2_, in order to facilitate the manufacturing process. When the thickness of overlay is adequately designed to produce a round-trip phase shift of 180°, the light waves reflected from the top and bottom interfaces are cancelled out via destructive interference, and as a consequence, the reflectivity of the structure is profoundly diminished.

The role of the dielectric overlay in acquiring ODR has been elaborated as well. The ODR was acquired by adaptively compensating for the deviation in the phase shift related to the resonant cavity, which changes with the incident angle. In regard to the angular dependence, the center wavelength shifted by about 18.9 nm, translating into 3.5%, while the normalized peak transmission was altered within 0.5 dB for incident angles ranging up to θ_o_ = 70° for the p-polarization. As mentioned earlier, the proposed scheme offers competitive performance in terms of the ODR, compared to the conventional approaches that are based on the MDM subwavelength grating, metallic nano-antenna array, and plasmonic nano-structure^12^,^13^,^14^,^15^,^16^. Those devices, which are mostly of reflective-type configuration^13^,^14^,^15^,^16^, were chiefly concerned about angle insensitive responses in the infra-red spectral bands^12^,^13^,^14^. The proposed color filter, providing comparable angular tolerance in the transmission-type configuration, is conspicuously advantageous in view of its high scalability, which is enabled by virtue of the simple fabrication process merely involving deposition of thin films, resorting to no subwavelength patterning^12^,^13^,^14^,^15^. Moreover, we inspected the dependence of the device performance on the light polarization in terms of the incident angle. The filters exhibit a much better angular tolerance for p-polarization than for s-polarization, which is beneficial when applied to devices such as liquid crystal displays. The undesirable polarization sensitivity may be readily mitigated by concocting a nano-resonator that exploits such a dielectric cavity as having a higher refractive index and a lower extinction coefficient in the visible band. Since the cavity can be drastically thinned so as to incur no significant propagation phase shift for both polarizations, the cavity rarely incurs polarization sensitivity^17^,^18^. It is noteworthy that despite the fact that TiO_2_ has a lower refractive index than a-Si in the visible band, the proposed omnidirectional color filters based on a nano-resonator could provide an appreciable improvement in terms of the angular tolerance and the transmission efficiency, with the assistance of the phase compensating dielectric overlay.

## Methods

### Simulation

The simulations to obtain the transmission/reflection spectra for normal and oblique incidence were conducted using Essential Macleod (Version 9.8.436), a commercially available tool specializing in the analysis of thin-film structures.

### Device fabrication

The proposed filters were manufactured on a glass substrate. Prior to the deposition of the thin films, organic and inorganic contaminants were successively removed from the substrate via ultra-sonification in acetone, ethanol, and deionized water. A 23-nm thick Ag film, a TiO_2_ cavity with different thicknesses of 100, 75, and 50 nm for the respective RGB filters, a similar Ag film with 23-nm thickness, and a 60-nm thick TiO_2_ overlay were subsequently deposited over an area of 7.63 × 2.54 cm^2^ via RF sputtering (AJA Sputter Coater System).

### Optical characterization

A cross-sectional structure of the fabricated blue filter was observed under a high resolution scanning electron microscope (UltraPlus analytical FESEM, Zeiss), as displayed in Figure 1(c). The thickness and the index of refraction of the grown films were checked using a reflecto-spectrometer (Filmtek4000, SCI) operating in a spectral range from 450 to 1,650 nm. To check the transmission spectra in the cases with normal and oblique incidence, a collimated light beam from a halogen lamp (Model LS-1, Ocean Optics) was irradiated upon the prepared device. The device was mounted on a motorized rotation stage, and the transmitted light was captured by a multimode fiber linked to a spectrometer (Model USB-4000-VIS-NIR, Ocean Optics). A calcite crystal polarizer (GTH 10M-A, Thorlabs) was used to polarize the incident light.

## Author Contributions

C.S. and V.R. are equally responsible for the design, optical characterization, analysis of the device, and writing the manuscript; S.S. supervised the analysis and co-wrote the manuscript; E.S. provided advice and support in preparing the manuscript; D.Y. fabricated the device, captured the SEM images, and measured the thickness and index of refraction of the deposited films. All authors discussed the results and implications and also commented on the manuscript at all stages.

## Supplementary Material

Supplementary Information Supplementary Information
