# Supplementary material for: Omnidirectional color filters capitalizing on a nano-resonator of Ag-TiO2-Ag integrated with a phase compensating dielectric overlay
Source: Sci Rep. 2015 Feb 16;5:8467. doi: 10.1038/srep08467 (PMC4329542; doi:10.1038/srep08467)
Supplement: Supplementary Information [file srep08467-s1.doc]

Supplementary Information

**Omnidirectional color filters capitalizing on a nano-resonator of Ag-TiO_2_-Ag integrated with a phase compensating dielectric overlay**

*Chul-Soon Park^1^, Vivek Raj Shrestha^1^, Sang-Shin Lee^1^,^*^ Eun-Soo Kim^1^, and Duk-Young Choi^2^*

^1^Department of Electronic Engineering, Kwangwoon University,

20 Kwangwoon-ro, Nowon-gu, Seoul 139-701, South Korea

^2^Laser Physics Centre, Research School of Physics and Engineering, Australian National University, Canberra ACT 0200, Australia

^*^E-mail: *slee@kw.ac.kr*

Figure S1 shows the refractive indices of Ag and TiO_2_ used for the calculations.

**
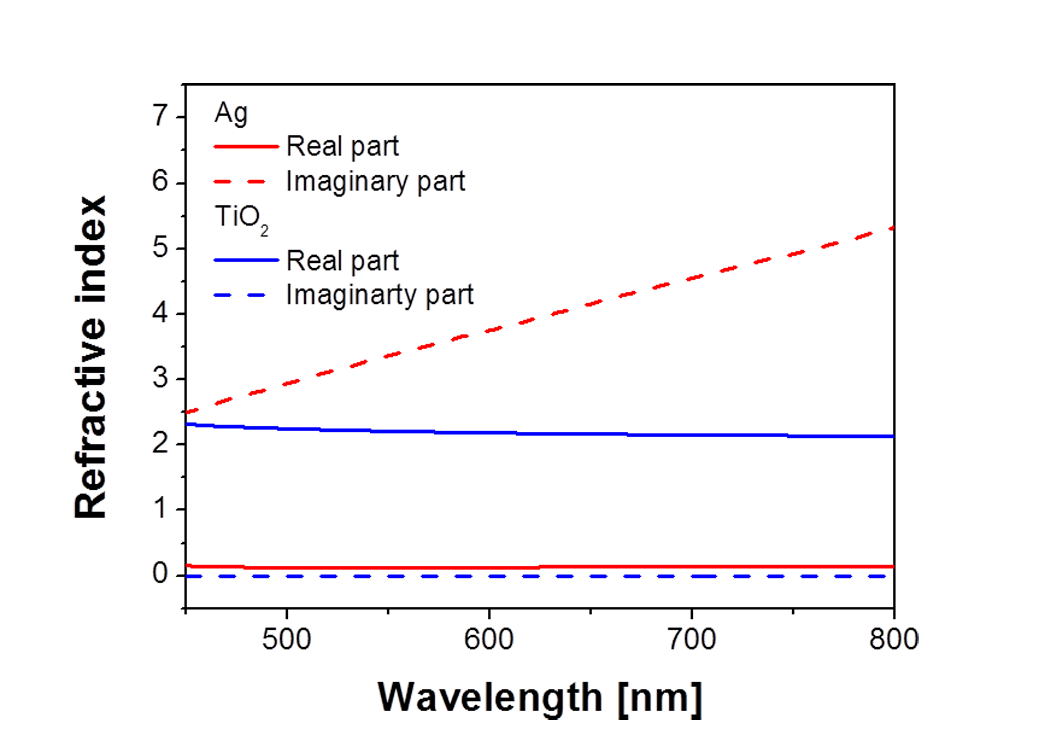
**

**Figure S1.** Refractive indices of Ag and TiO_2_ adopted for simulation. The data for TiO_2_ were obtained from ellipsometric measurements of deposited films while that of Ag was obtained from [1].

**
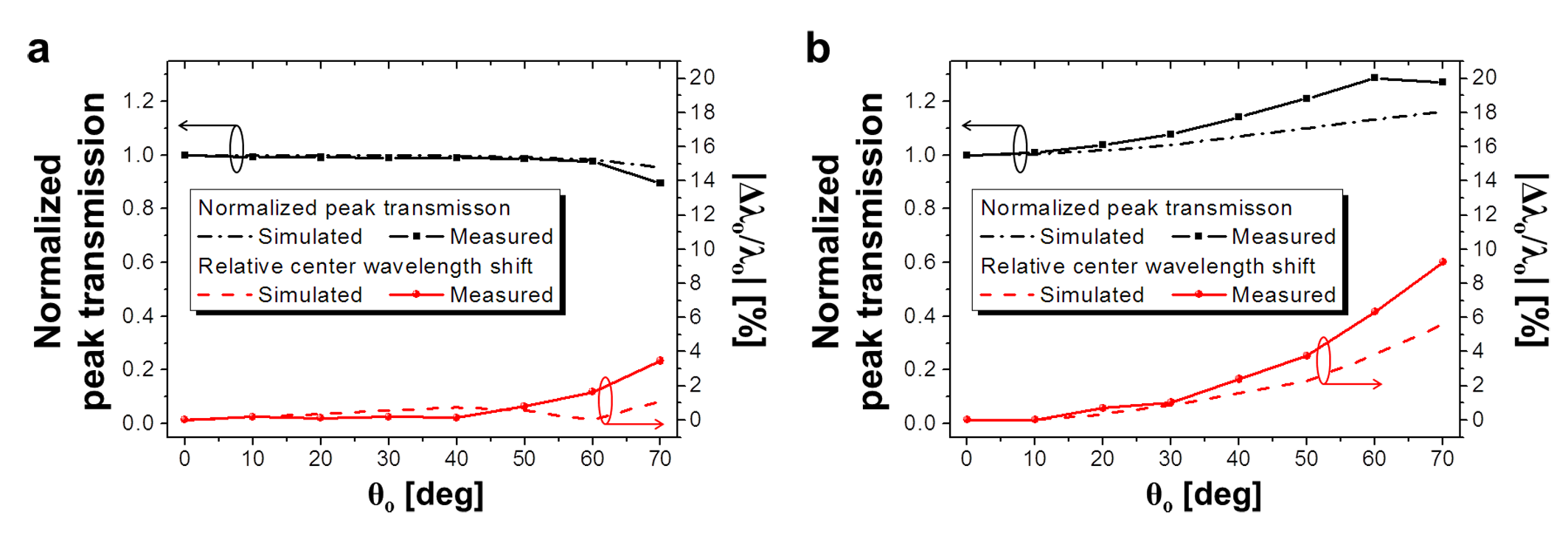
**

**Figure S2.** Simulation and measurement results for the normalized peak transmission and relative center wavelength shift for the green filter with (a) (Ag-TiO_2_-Ag)|TiO_2_ and (b) Ag-TiO_2_-Ag structures, respectively.

The calculated total phase differences, defined as , where the and are the reflection phase shifts at the top and bottom Ag-TiO_2_ cavity interfaces; the round-trip propagation phase shift, in Ag-TiO_2_-Ag resonator without and with a TiO_2_ overlay are shown in Figures S3 and S4, respectively. The total phase difference implying the variation of resonant wavelength is varying even for the p-polarization for the Ag-TiO_2_-Ag structure, compared to the (Ag-TiO_2_-Ag)|TiO_2_ structure. From the phase compensation, the nano-resonator with a dielectric overlay can deliver omnidirectional transmission spectra under p-polarization.


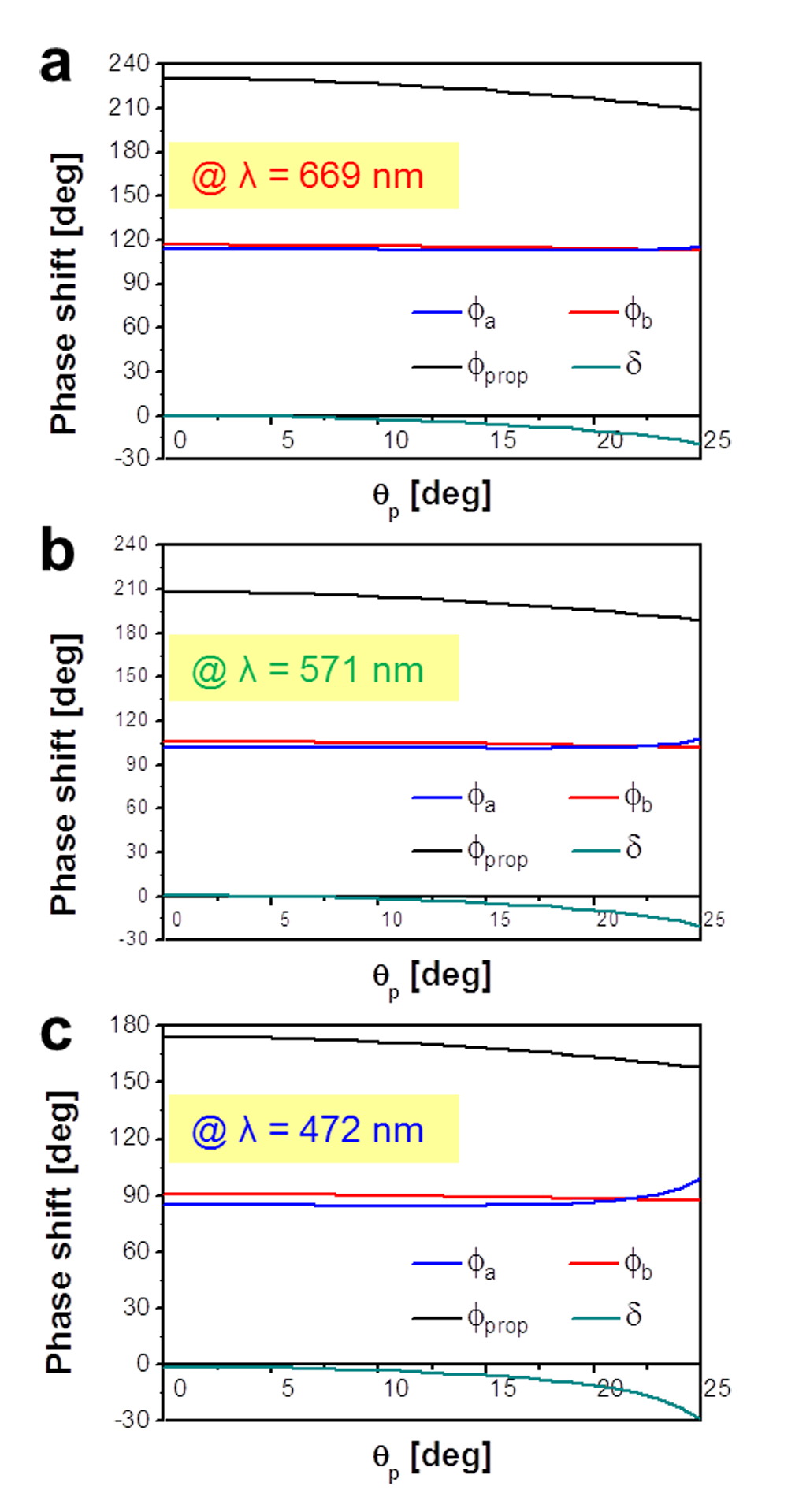


**Figure S3.** Calculated total phase difference for the Ag-TiO_2_-Ag nano-resonator as a function of θ_p_ ranging from 0º to 25º for p-polarized incident light.


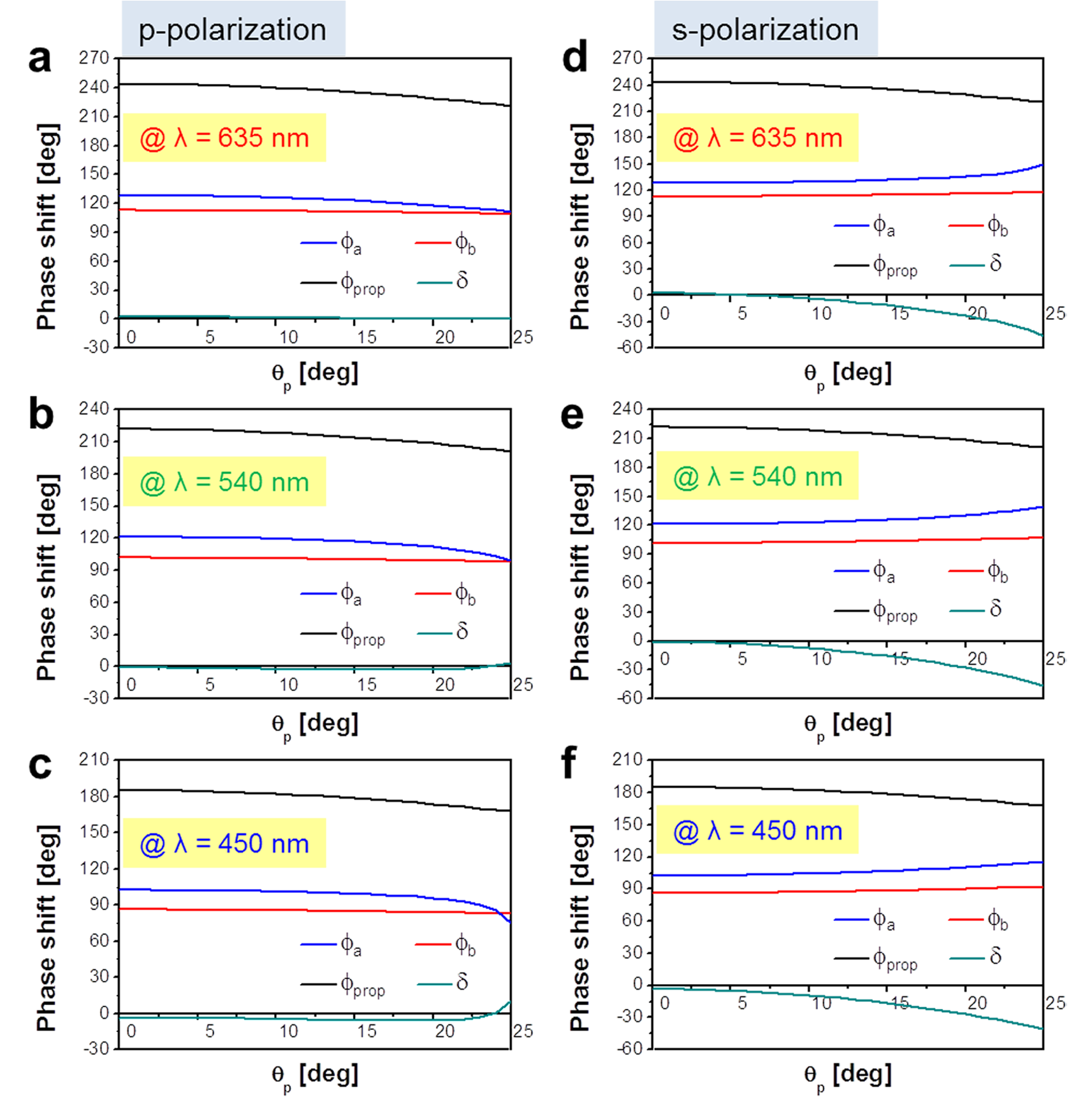


**Figure S4.** Calculated total phase difference in a (Ag-TiO_2_-Ag)|TiO_2_ nano-resonator, which capitalizes on a phase compensating overlay as a function of θ_p_ ranging from 0º to 25º (a-c) for the p-polarization and (d-f) for the s-polarization.


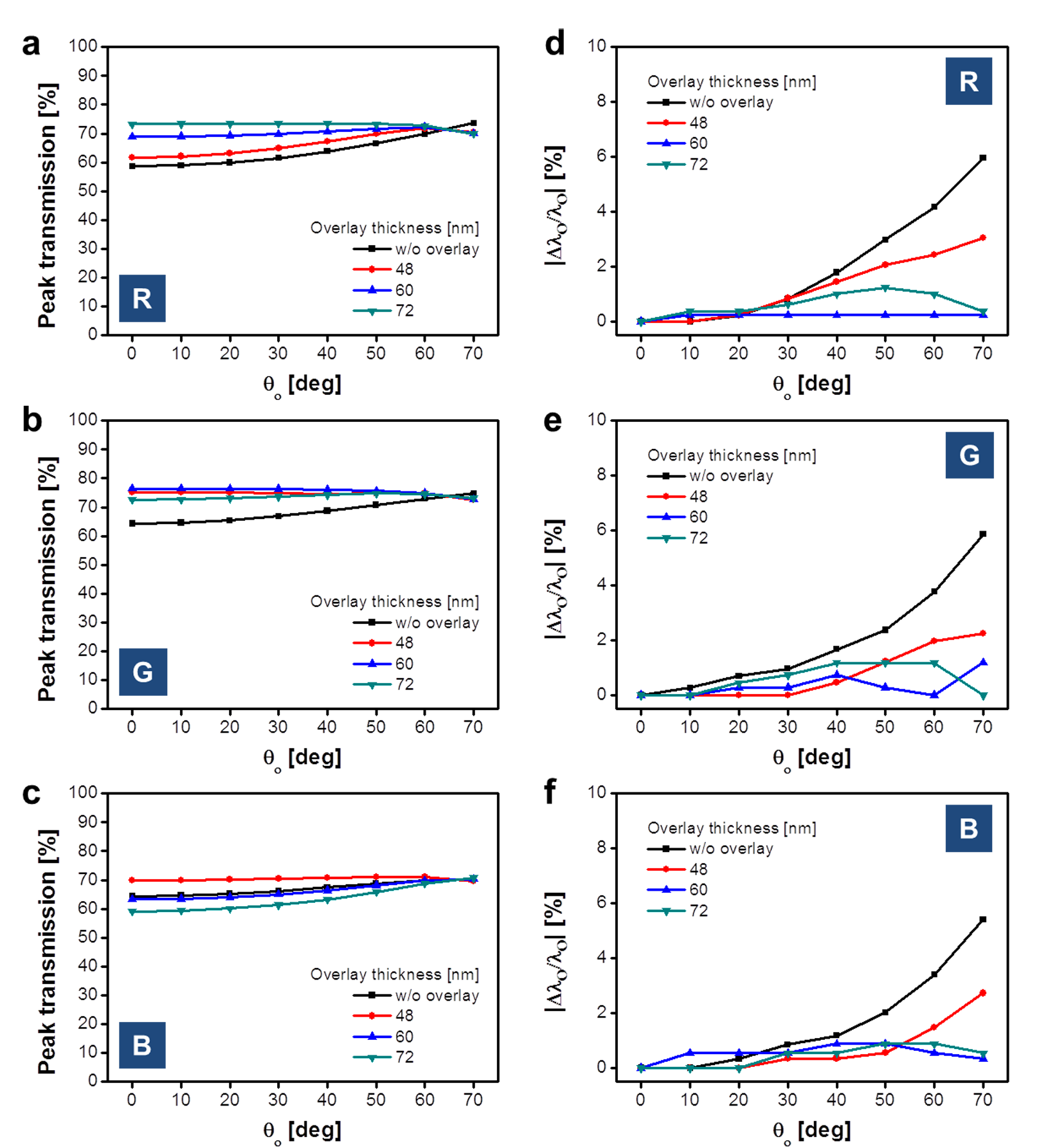


**Figure S5.** (a-c) Calculated peak transmission and (d-f) relative center wavelength shift with the incident angle in terms of the thickness of the dielectric overlay, for the RGB filters.

**References**

1. Palik, E. D. *Handbook of Optical Constants of Solids III* (Academic Press, San Diego, USA, 1998).
